# Baseline Stability of Thermally Hydrosilated Porous Silicon with Zwitterionic Antifouling Polymer Coating for Biosensing Applications

**DOI:** 10.1021/acsomega.5c03495

**Published:** 2025-07-16

**Authors:** Soren M. Smail, Paul E. Laibinis, Sharon M. Weiss

**Affiliations:** † Interdisciplinary Materials Science Graduate Program, 5718 Vanderbilt University, Nashville, Tennessee 37235, United States; ‡ Department of Chemical and Biomolecular Engineering, Vanderbilt University, Nashville, Tennessee 37235, United States; § Department of Electrical and Computer Engineering, Vanderbilt University, Nashville, Tennessee 37235, United States

## Abstract

Porous silicon (PSi)-based biosensors are a promising platform for quantitative rapid diagnostics, but they have not broadly realized clinically relevant limits of detection due, in part, to poor baseline stability. Baseline instability can be attributed to two major physicochemical challenges—hydrolysis of PSi in aqueous solutions and fouling by unwanted biological species, both of which can obscure the detection of target molecules at low concentrations. In this work, PSi was thermally hydrosilated with vinylbenzyl chloride (VBC) to incorporate hydrolytically stable Si–C bonding and to provide an attached alkyl halide termination for further chemistry. Subsequent grafting of zwitterionic poly­(sulfobetaine methacrylate) (SBMA) from this PSi-VBC layer by surface-initiated atom-transfer radical polymerization (siATRP) formed an antifouling coating. Films both with and without the antifouling polymer were exposed to PBS (pH 7.4) and human blood serum, and optical reflectance measurements were used to monitor hydrolysis and nonspecific adsorption. PSi-VBC-polySBMA surfaces exhibited little to no nonspecific binding, as determined by ATR-FTIR and optical reflectance measurements, due to their hydrophilicity. The compatibility of hydrosilylation and siATRP with various chemical groups provides significant versatility in this surface chemistry approach, as well as facilitates the incorporation of highly specific capture agents. By directly addressing the issues of hydrolysis and fouling, this strategy holds promise for reducing the limits of detection in complex biological samples.

## Graphical abstract

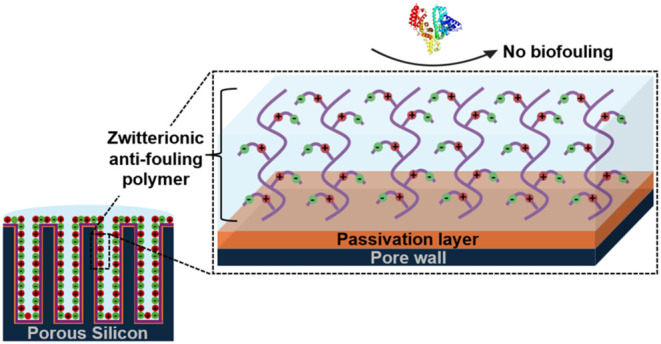

## Introduction

Porous silicon (PSi) offers a promising and versatile platform for optical biosensing due to its simple fabrication process, compatibility with various surface chemistries, and its ease of measurement of a change in structural color.^1^ Specifically, the capture of target biomolecules within PSi produces a change in the effective refractive index of the PSi film, such that the amount of material captured within the PSi layer is quantifiable by straightforward reflectance measurements in a label-free manner. By this approach, PSi-based optical sensors have been reported for the detection of a wide variety of species including oligonucleotides,^2–4^ proteins,^5–7^ and bacteria.^8,9^ However, to achieve clinical relevance, further progress must be made to enable PSi biosensors to reliably attain limits of detection comparable to those of gold-standard diagnostics such as ELISA and PCR.^1,10–13^ Accordingly, two key challenges that hinder accurate molecular detection at low analyte concentrations must be addressed: native PSi surface degradation in aqueous solutions and biofouling from nontarget molecules.

Negative baseline shift due to hydrolysis of PSi has been an ongoing issue for biosensing applications, and extensive work has been done to passivate PSi surfaces through methods such as oxidation, carbonization, and hydrosilylation,^14,15^ each of which can allow further functionalization. The most used passivation method is oxidation, which provides improved stability in aqueous solutions over hydride-terminated PSi, but problems of hydrolysis remain.^16–18^ Alternative methods that form robust Si–C bonds to the surface of PSi have been developed. One such method, thermal carbonization, has been accomplished by thermal decomposition of acetylene to form a protective carbon-containing layer.^19^ While carbonized PSi surfaces have shown excellent stability in aqueous solutions and offer the ability of postfunctionalization,^20–23^ this method requires specialized equipment to provide the necessary reaction conditions. Another method of forming Si–C bonds, hydrosilylation, can be accomplished in a straightforward manner by thermal or photochemical addition of an unsaturated hydrocarbon.^24^ Hydrosilylation has been demonstrated to be compatible with various end-group functionalities, enabling subsequent surface chemistries.^25–27^ However, previous reports of hydrosilated PSi suggested that this approach for achieving Si–C bonds to the surface was not sufficiently robust to prevent hydrolysis.^21,22^

Fouling by unwanted biomolecules is a widespread issue for all label-free biosensing, as signals from nontarget molecules can lead to false positive diagnostic results and significantly impede achieving low limits of detection.^28^ The development of antifouling polymers for reduced protein adsorption has been demonstrated in a number of applications,^29,30^ but their use on PSi platforms has been limited.^31–33^ For PSi-based sensors, nonspecific adsorption has been mitigated by use of blocking agents (e.g., bovine serum albumin) or the attachment of polyethylene glycol (PEG) compounds following biofunctionalization with highly specific capture agents.^34–37^ While PEGs are a common choice of antifouling polymer, they can deteriorate in aqueous solutions,^29,38,39^ diminishing their antifouling properties. For PSi-based optical sensors, the deterioration of an antifouling coating may lead to negative baseline shift. In other areas, zwitterionic polymers have gained attention as effective alternatives to PEGs for providing antifouling properties due to their hydrolytic stability and notable performance, the latter an effect of forming a strongly bound hydration layer to these highly charged yet net-neutral materials.^40–42^ To date, the grafting of zwitterionic polymers from a PSi-based biosensor platform has not been demonstrated. Atom-transfer radical polymerization (ATRP)^43^ provides a widely used method for the preparation of zwitterionic polymers, both in solution and as coatings from surfaces. Its exceptional compatibility with a wide variety of chemical groups enables the formation of polymers with diverse compositions and functionalities.^43–49^ Functional sites for coupling biomolecules and other species (i.e., highly specific capture agents) can be introduced using monomers with intrinsic reactivity as components of the polymer.^40^ For ease of preparation of the zwitterionic coatings, an analog of ATRP can be used that avoids the rigorous deoxygenation requirements of ATRP. This method, activators regenerated by electron transfer (ARGET)-ATRP, has been developed to include a reducing agent, for more operational ease-of-use.^50^ Importantly, because the common approach utilized to form zwitterionic polymers only requires alkyl halide surface termination, it is possible to graft a zwitterionic polymer from PSi that could serve to both anchor capture agents such as antibodies and prevent nonspecific binding of unwanted molecules.

In this work, we determine appropriate conditions for thermal hydrosilylation of vinylbenzyl chloride (VBC) onto PSi to enable the formation of a Si–C anchored film that can promote stability to PSi against corrosion in aqueous solutions and provide the necessary alkyl halide termination for subsequent grafting of a zwitterionic poly­(sulfobetaine methacrylate) (polySBMA) antifouling coating by ARGET-ATRP. We show that hydrosilylation temperature plays a key role in the resulting surface coverage of VBC and the robustness of the modified PSi surface in aqueous solutions. Under the identified conditions, there is minimal corrosion of the PSi over time durations relevant for biosensing applications. We demonstrate the ability of VBC-modified PSi to provide initiation sites for ARGET-ATRP opening this route for PSi modification. By this approach, we generate a grafted zwitterionic polymer from the VBC-modified PSi and show that this antifouling coating prevents nonspecific binding when the PSi film is exposed to human serum. By addressing issues of PSi surface stability in aqueous solutions and nonspecific binding in a biological fluid, VBC-initiated polySBMA films on PSi offer a compelling surface on which to further develop label-free PSi-based biosensors capable of highly sensitive and highly specific molecular detection in complex fluids.

## Experimental Section

### Materials

4-Vinylbenzyl chloride (VBC, 90%), [2-(methacryloyloxy)­ethyl]­dimethyl-(3-sulfopropyl)­ammonium hydroxide “sulfobetaine methacrylate” (SBMA), copper­(II) bromide, methanol (MeOH), ethanol (EtOH), and acetone were purchased from Sigma-Aldrich. Tris­(2-pyridylmethyl)­amine (TPMA) and l-ascorbic acid were purchased from TCI America. Hydrofluoric acid (HF, aq., 48–51%) and phosphate buffered saline (PBS) pH 7.4 were purchased from Thermo Scientific. Single-side polished, boron-doped p-type silicon wafers (⟨100⟩, 0.01–0.02 Ω-cm, 500–550 μm) were purchased from Pure Wafer. Pooled off-the-clot human serum was purchased from Innovative Research, Inc. All chemicals were used as received. Deionized (DI) water was produced using a Millipore Elix water purification system (resistivity 15 MΩ-cm).

### PSi Fabrication

Silicon wafers were first electrochemically etched in a 3:7 HF/EtOH solution at a current density of 90 mA/cm^2^ for 50 s to form a sacrificial PSi layer. Wafers were subsequently washed with water and dried with N_2_, then submersed in 1 M NaOH (1:4 DI water/EtOH) to remove the sacrificial layer. A second electrochemical etch, identical to the first one, was then performed, and the etched wafers were subsequently washed with DI water and dried with N_2_. PSi wafers were diced into 4 mm × 4 mm chips before use.

### Thermal Hydrosilylation

PSi was immersed in 2.5% aq. HF solution for 90 s to remove native oxides, washed with DI water and EtOH, dried with N_2_, and then sealed in vials where the headspace was purged with N_2_ for 5 min to displace ambient oxygen. VBC that had been sparged for 30 min with N_2_ was syringed into PSi-containing vials that were then placed into a preheated oil bath of 70, 85, or 100 °C. After desired reaction times, samples were cooled to room temperature and soaked in acetone for 8 h to remove unbound VBC.

### ARGET-ATRP of SBMA

A 500-μL aliquot from a solution formed by dissolving CuBr_2_ (40 mg) in 10 mL of MeOH was combined with TPMA (12.5 mg). Next, 60 μL of the CuBr_2_/TPMA solution was added to a solution comprising 75 mg of SBMA, 8 mL of DI water, and 7 mL of MeOH, and the resulting solution was sparged with N_2_ for 30 min. A 1.2 mL aliquot of a l-ascorbic acid solution formed by sonicating l-ascorbic acid (100 mg) in 4 mL of MeOH for 15 min was added to the CuBr_2_/TPMA/SBMA solution, and the resulting solution was sparged with N_2_ for 5 min. Thermally hydrosilated samples were sealed in vials, the headspace sparged with N_2_ for 5 min to displace ambient oxygen, and then sparged CuBr_2_/TPMA/SBMA solution was added by syringe. After 16 h of immersion, PSi samples were washed in DI water/MeOH (1:1 by volume) on a shaker table for 16 h, rinsed sequentially with DI water and MeOH, and then dried with N_2_.

### Optical Measurements

Reflectance measurements of PSi were obtained using a Thorlabs SLS201L tungsten halogen light source coupled into a bifurcated fiber reflectance probe (Ocean Optics). Reflected light was collected by the reflectance probe and coupled into an Ocean Optics USB 4000 CCD spectrometer. Ocean Insight Ocean Direct software was used to measure an average of 200 scans over an integration time of 10 ms. The effective optical thickness (EOT) of each sample was calculated using the reflective interferometric Fourier transform spectroscopy (RIFTS) method.^51^ For real-time in situ experiments, a multichannel fluidic cell was used.^52^ O-rings were placed over PSi samples that were then secured between two plexiglass sheets, one supporting the back of the sample and the other providing optical access to the PSi layer. The plexiglass setup with PSi samples was mounted on a Python script-driven stepper motor to facilitate staggered measurements of multiple samples at ∼40 s intervals. Inlet and outlet apertures in the plexiglass were used to supply 20 μL of PBS (pH 7.4) to each PSi sample. Reflectance measurements of the PSi samples were obtained before, during, and after exposure to PBS.

### ATR-FTIR

Attenuated total reflection Fourier transform infrared (ATR-FTIR) spectra were obtained using a Nicolet 6700 IR spectrometer equipped with a Thermo Scientific Smart iTR ATR sampling accessory. An average of 256 scans at a resolution of 2 cm^–1^ were acquired for each spectrum in absorbance mode with a background of air.

### Stability Experiments

#### Hydrolytic Stability

PSi with varying levels of surface coverage by VBC (both with and without a polySBMA coating) were immersed in PBS (pH 7.4) and placed on a shaker table for specific time intervals. Samples were rinsed sequentially with DI water and EtOH and then dried with N_2_ before each reflectance measurement. Three measurements from ≥4 samples of each surface modification were obtained and averaged.

#### Nonspecific Adsorption

PSi-VBC and PSi-VBC-polySBMA were exposed to PBS (pH 7.4) or human blood serum for 90 min and then washed in PBS (pH 7.4) on a shaker table for 30 min to remove unbound proteins. Samples were rinsed with DI water, then dried with N_2_ before measurement.

## Results and Discussion

Figure 1 provides an overview of our modification process to yield a layer of PSi coated internally with covalently anchored zwitterionic polymer films. First, silicon wafers were electrochemically etched in a hydrofluoric acid–based electrolyte to produce on-substrate PSi films of approximately 2 μm in thickness with an average pore size of 30 nm, as estimated based on analysis of scanning electron microscopy images (Figure 1a). Next, these hydride-terminated PSi films were thermally hydrosilated with VBC to form an organic film having robust Si–C bond attachments and available chloride end groups to serve as initiator sites for ATRP. Lastly, polySBMA brushes were grafted from PSi-VBC using ARGET-ATRP, forming an approximately 5 nm thick zwitterionic coating to provide antifouling properties. The polySBMA thickness was estimated by ellipsometry from experiments on silicon wafers that were modified under the same thermal hydrosilylation and SBMA grafting conditions as with PSi samples.

**Figure 1:**
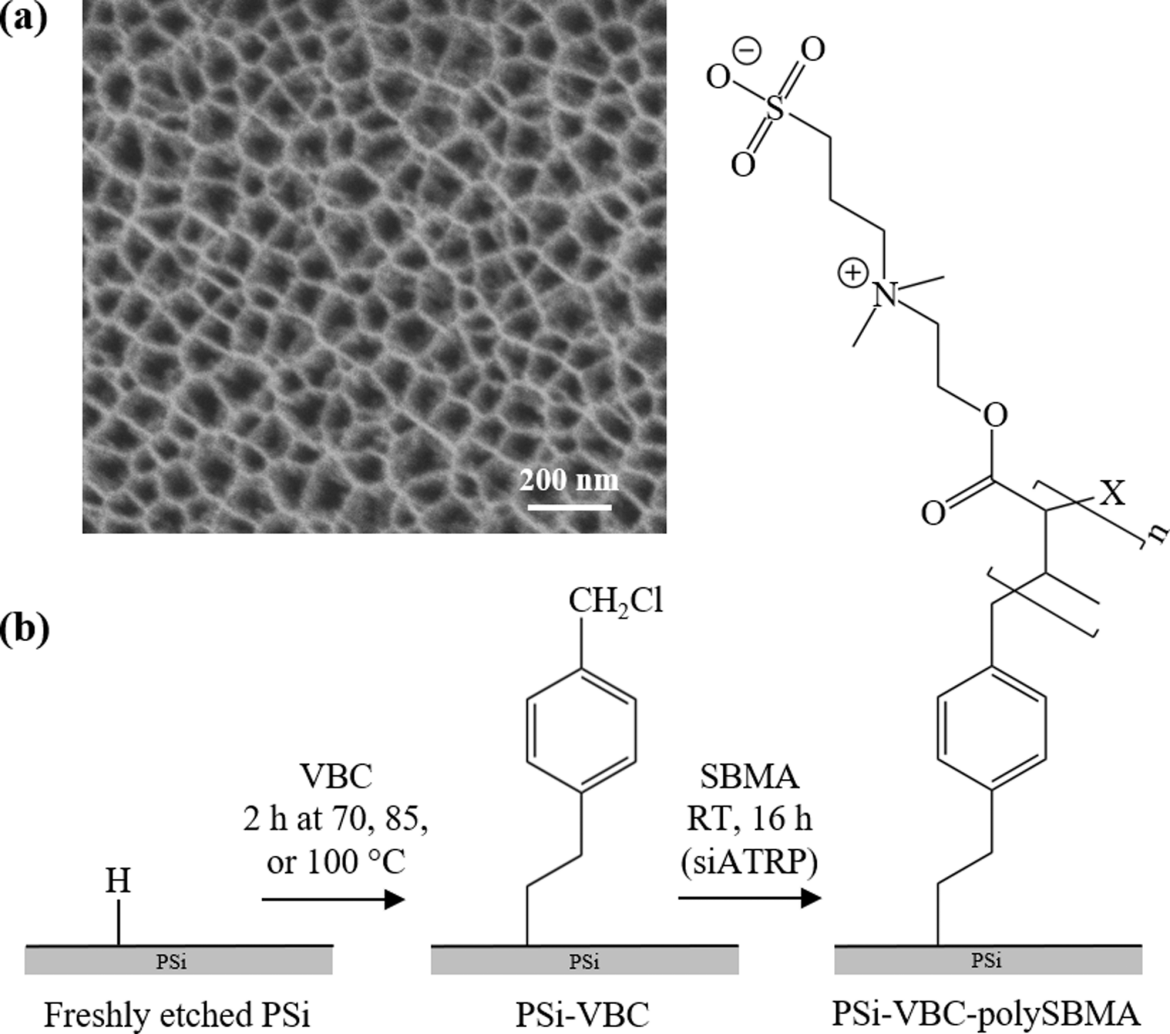
(a) Top-view SEM image of PSi surface. (b) Two-step surface modification to PSi: Freshly etched PSi is thermally hydrosilated with VBC, then formation of a grafted polySBMA film via ARGET-ATRP. Side chains of zwitterionic polySBMA contain positively and negatively charged groups, and the chain end is terminated by a halide, denoted as X.

To validate successful surface attachment of VBC and polySBMA, we collected ATR-FTIR spectra after each step in the process. Following thermal hydrosilylation of freshly etched PSi films with VBC, the spectra showed a reduction in silicon hydride peak (2100 cm^–1^) intensity from that on the native PSi surface (Figure 2a), compatible with conversion of Si–H to Si–C bonds. In other areas of the spectra, the appearance of peaks corresponding to ring overtones from aromatic species (1500 cm^–1^), stretching modes for C–H bonds (2840 and 2910 cm^–1^), and C–H wag absorption for terminal halides (1260 cm^–1^) support the attachment of a benzyl chloride moiety to the PSi. To compare the effects of hydrosilylation reaction temperature on VBC attachment, we replicated experiments across four samples at each reaction condition and examined intensity differences in the C–H wag mode for the CH_2_Cl moiety in the IR spectra. To account for variations in the contact between PSi samples and the ATR crystal that can affect spectral intensity, we normalized the maximum peak (1020 cm^–1^) intensity in each spectrum to a common value and then integrated the CH_2_Cl peak intensity. PSi samples hydrosilated in neat VBC at 85 and 100 °C exhibited terminal halide peak areas that were ∼30 and ∼140% greater than those for PSi hydrosilated at 70 °C, respectively (Figure S1a). These results indicate that higher reaction temperatures lead to increased surface coverage by VBC, providing the possibility for more sites for initiating ATRP reactions.

**Figure 2:**
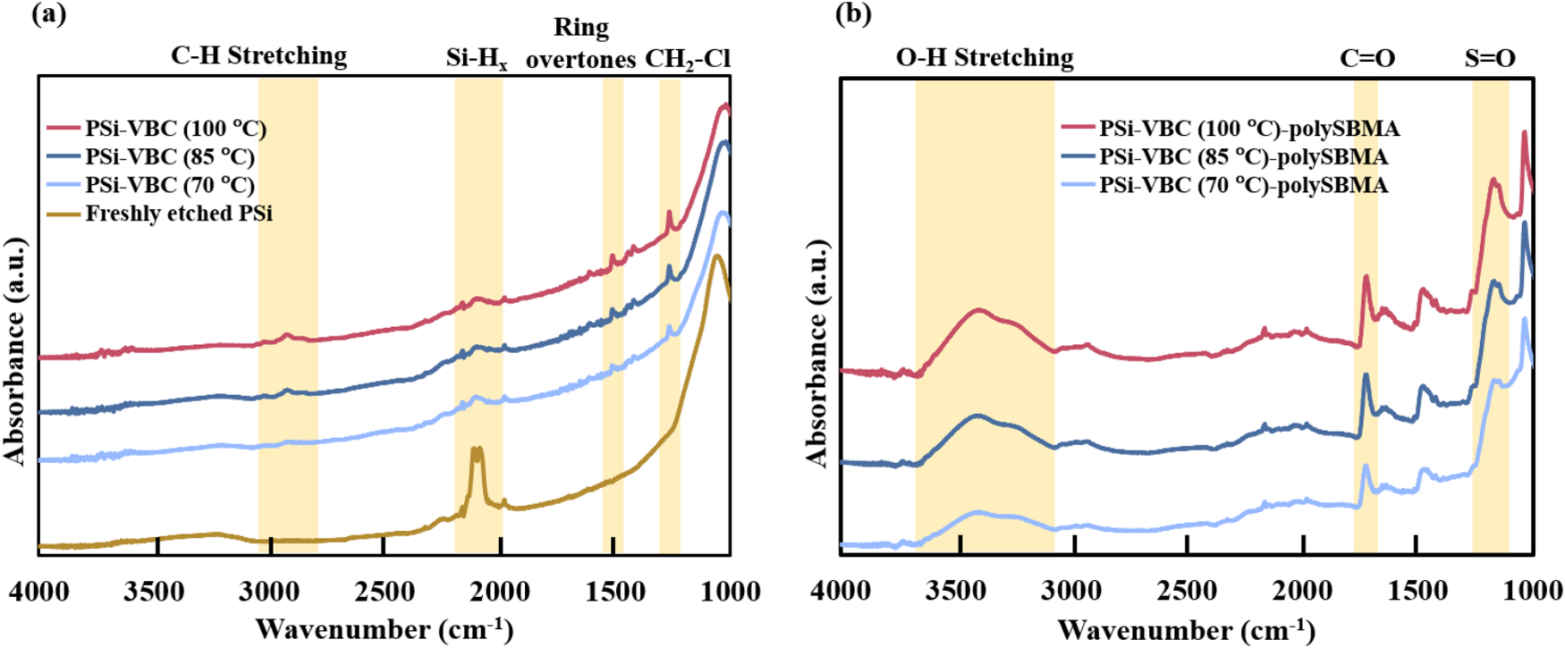
ATR-FTIR spectra of (a) PSi as-prepared and after hydrosilylation with VBC at 70, 85, and 100 °C and (b) VBC-modified PSi after ARGET-ATRP to form a polySBMA layer. Key spectral regions are highlighted and discussed in the main text. Spectra are offset vertically for clarity.

In a second step, the PSi-VBC samples underwent ARGET-ATRP with SBMA as the monomer. Grafting of SBMA is confirmed in Figure 2b by the presence of peaks for C=O (1725 cm^–1^) and S=O (1175 cm^–1^) bonds. Moreover, the appearance of a broad O–H stretching peak from 3100 to 3700 cm^–1^ indicates the hygroscopic nature of the polySBMA-coated surface, being due to a strongly held hydration layer. The appearance of these new peaks in the spectra for the PSi samples with polySBMA occur with a near but not complete loss of the peak at 1260 cm^–1^ from the benzyl chloride group used for ARGET-ATRP initiation from the surface.

To assess differences in the polySBMA surface coverage as affected by the VBC hydrosilylation reaction conditions, we compared ATR-FTIR spectra of four replicates of PSi samples modified with VBC at each hydrosilylation condition (70, 85, and 100 °C) that then underwent SBMA grafting by ARGET-ATRP under the same polymerization conditions. In Figure 2b, the peaks at 1175 and 1725 cm^–1^ assigned to polySBMA show an increasing intensity on surfaces modified with VBC at the higher temperatures, that is, those that exhibit higher coverages of attached VBC. Specifically, samples hydrosilated with VBC at 85 and 100 °C had C=O peak areas from polySBMA that were 53 and 66% greater than those of samples hydrosilated at 70 °C, respectively, (Figure S1b). These increases indicate greater formation of grafted polySBMA on the PSi films that had greater VBC coverage (i.e., those formed at the higher reaction temperatures). This result is likely due to those surfaces providing more terminal halides to serve as initiation sites for ATRP. While these higher coverages of VBC produced greater amounts of polySBMA, they also led to more unreacted terminal halide initiator sites that remained on each surface, as seen in Figure 2b. These sites likely became inaccessible as a higher density of growing polySBMA chains blocked initiation sites, suggesting high coverage of polySBMA on the PSi.

The VBC hydrosilylation reaction conditions that led to increased polySBMA also resulted in higher levels of absorbed water as surfaces with greater C=O peak areas also had greater OH peak areas. Specifically, samples hydrosilated at 85 and 100 °C had OH peak areas that were 69 and 126% greater than those of samples hydrosilated at 70 °C, respectively (Figure S1c). Increased water content is desired for achieving antifouling properties as the hydration layer can retard adsorption of many classes of biomolecules.

As PSi hydrosilated with VBC at higher temperatures provided greater coverage by both VBC and SBMA, making them more desirable, we examined even higher reaction temperatures. At higher reaction temperatures (≥120 °C), we observed that VBC would uncontrollably polymerize, block the pores, and not allow grafting of the antifouling polymer by ATRP. In some cases, the VBC-modified PSi layer would retain Fabry–Perot fringes (Figure S2a) and the ATR-FTIR spectra would show the expected peaks; however, subsequent grafting of SBMA to this surface was unsuccessful (Figure S3). For these surfaces, it is likely that the pores filled or became blocked by VBC. We generally observed that with extended reaction times and higher temperatures, the polymerization of VBC formed a thick, nonuniform gel on top of the PSi layer, eliminating the appearance of Fabry–Perot fringes in reflectance spectra, making them unsuitable for sensing (Figure S2). If surface coatings providing the desired functionalities (i.e., hydrolytic stability and antifouling properties) become too thick, they can impede molecular infiltration and negatively affect detection limits. Importantly, we also observed that the optimal reaction time and temperature for achieving protection against hydrolysis and fouling can vary with VBC vendor and lot number, possibly due to differences in impurities and inhibitors. The reported results are those we observed most consistently.

To assess the ability of PSi-VBC and PSi-VBC-polySBMA samples to resist hydrolysis, samples were immersed in PBS (pH 7.4) for specific time intervals, and changes in EOT determined from reflectance measurements. Based on the ATR-FTIR analysis, we expected that samples that underwent hydrosilylation at higher temperatures would exhibit improved protection against hydrolysis due to having a greater degree of conversion of surface Si–H bonds to Si–C bonds.^53^ Indeed, as shown in Figure 3 (solid lines), samples hydrosilated at the lower temperature of 70 °C experienced significantly more corrosion (ΔEOT = −0.34% after 1 h) than those hydrosilated at 85 and 100 °C (ΔEOT = −0.10 and −0.04% after 1 h, respectively). Accordingly, a high degree of conversion of Si–H to Si–C bonds during the passivation process is essential to minimize hydrolysis. With a goal to produce passivated PSi samples that could provide surfaces with antifouling properties toward biological fluids, duplicate hydrosilated sample sets from each reaction temperature underwent a subsequent grafting of polySBMA. As expected, PSi-VBC-polySBMA surfaces (dashed lines) with greater VBC coverage exhibited the greatest stability; however, when comparing PSi surfaces with and without polySBMA coating, films with a polySBMA coating exhibited more corrosion. This difference can largely be attributed to the hydrophilic polySBMA coating that provides increased pore access by water as opposed to hydrophobic PSi-VBC surfaces. The differences in hydrophilicity were dramatic as the water contact angle on PSi-VBC decreased from 90 to 12° by addition of the polySBMA film. While the parent PSi-VBC surfaces exhibited greater stability in PBS than their resulting PSi-VBC-polySBMA surfaces, the former do not provide the hydrophilicity desired for biosensing applications, as fouling by nonspecific proteins and analyte transport to capture sites must be considered.

**Figure 3:**
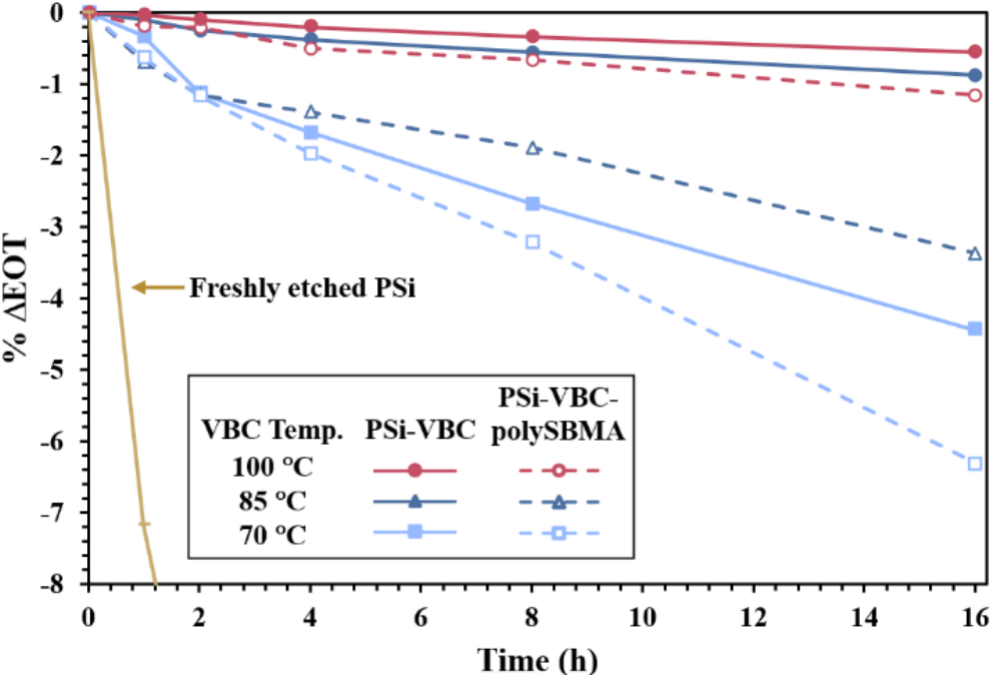
Relative change in EOT of PSi-VBC (solid lines) and PSi-VBC-polySBMA (dashed lines) films as determined from reflectance measurements in air after specific immersion times in pH 7.4 PBS. VBC films were formed at different reaction temperatures, and subsequent polySMBA films formed under common conditions.

To further confirm the hydrophilicity of SBMA and its consequences for PSi biosensors, we conducted real-time in situ reflectance measurements on PSi samples with and without polySBMA that were exposed to an aqueous PBS solution (Figure 4). The addition of polySBMA dramatically enhances water infiltration into the pores, as the aqueous PBS solution fills the pores of PSi-VBC-SBMA almost instantaneously (<1 min), whereas even 12 h after exposure to PBS, the pores of hydrophobic PSi-VBC had not completely filled. We note that the difference in the % ΔEOT is expected to be larger for PSi-VBC samples compared to PSi-VBC-polySBMA samples, as well as for samples hydrosilated at 85 °C compared to those at 100 °C, due to the larger void space available for PBS infiltration.

**Figure 4:**
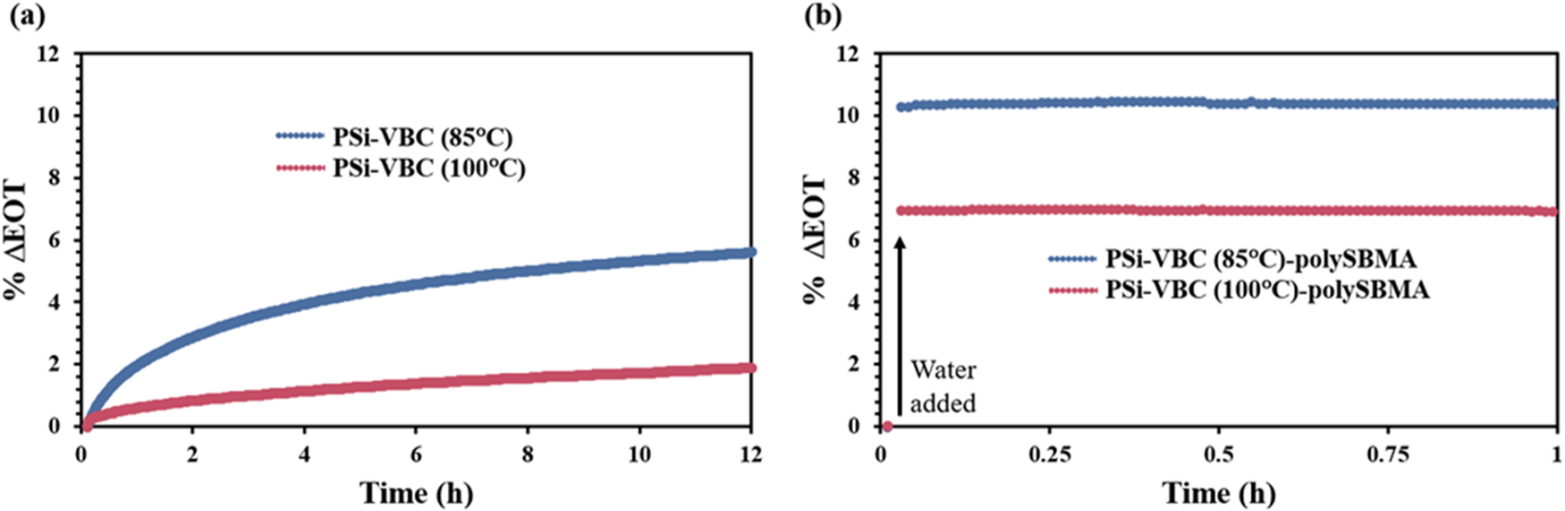
In situ reflectance measurements showing pore-filling time of PSi films upon exposure to pH 7.4 PBS: (a) hydrophobic PSi-VBC and (b) hydrophilic PSi-VBC-polySBMA films formed at two different VBC reaction temperatures.

The in situ reflectance measurements in Figure 4 showing dramatic differences in the times for an aqueous solution to fully contact the PSi inner surface suggest that the greater hydrolytic stability provided to the PSi-VBC samples without polySBMA (Figure 3) is due to limited water contact within the PSi film. For applications where protection against hydrolysis is the primary objective, hydrophobicity (and with it a capillary pressure that inhibits fluid intake into pores) can effectively resist corrosion. However, for biosensing purposes, hydrophobicity negates the advantage of PSi’s large surface area, as it prevents water from accessing the porous structure and capture agents immobilized on its surface. Because of the high aspect ratio of PSi pores, sensor response time is not only limited by molecular binding kinetics, but also by mass transport of analytes. With the incorporation of a hydrophilic coating on the PSi sensor surface, target analytes have improved access to capture agents, allowing for a larger signal from binding events to be achieved in shorter times. The dramatic contrast (seconds vs hours) between the rapid pore-filling of polySBMA-coated PSi and the slow pore-filling of hydrophobic PSi-VBC demonstrates the role of surface chemistry in influencing mass transport dynamics in PSi.

Finally, to verify the antifouling ability of the polySBMA films when on PSi, PSi-VBC-polySBMA samples were exposed to human blood serum, and changes in the EOT of the porous layer were monitored by optical reflectance measurements. As proteins (*n* = ∼1.5) have a higher refractive index than ambient air (*n* = 1.0), the effective refractive index of the porous layer will increase if proteins are added to the pores. Conversely, if material is removed from the pores, such as due to corrosion, the effective refractive index of the layer will decrease. Samples both with and without the antifouling polymer were incubated in serum for 90 min, then rinsed in PBS (pH 7.4) for 30 min to remove nonadsorbed proteins. As a control, duplicate samples were exposed to only PBS for a total of 120 min. When exposed to serum, PSi-VBC films without an antifouling polymer exhibited a large increase of 2.9% in EOT (Figure 5), indicating that nonspecific binding of proteins occurred. ATR-FTIR spectra of PSi-VBC samples after exposure to serum showed new peaks from amide I and II bands (1650 and 1550 cm^–1^), consistent with the presence of proteins (Figure S4). In contrast, samples with polySBMA experienced a shift of −0.10% EOT when exposed to serum, demonstrating a dramatic decrease in the level of fouling. The decrease in EOT of these samples was consistent with the small amount of hydrolysis experienced by control PSi-VBC-polySBMA samples that were exposed to only PBS. ATR-FTIR spectra of PSi-VBC-polySBMA samples after exposure to serum did not show evidence of amide I and II bands.

**Figure 5:**
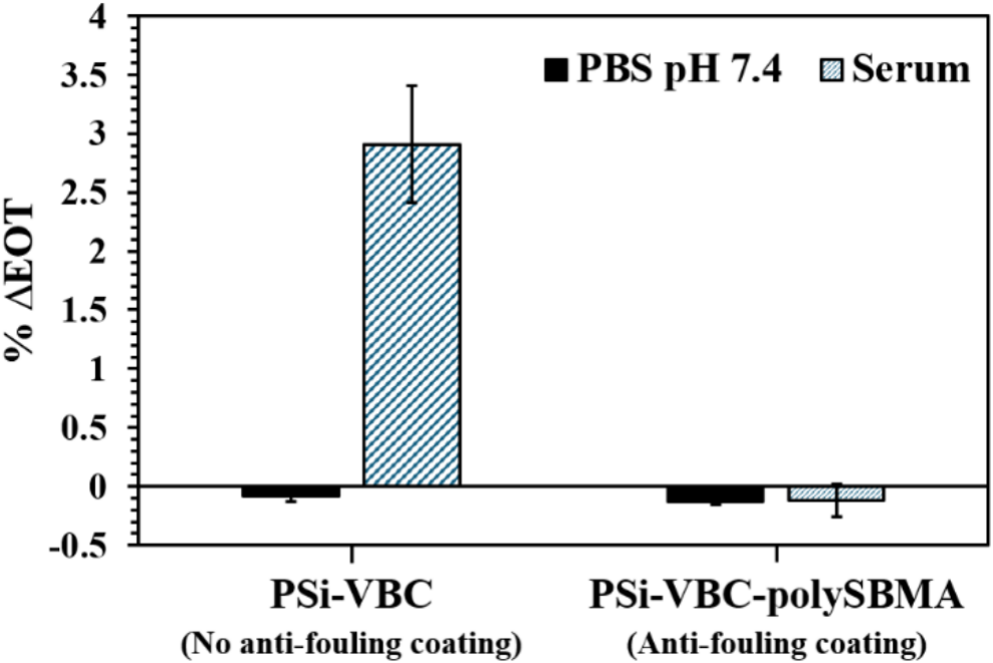
Reflectance measurements showing change in EOT of PSi-VBC and PSi-VBC-polySBMA films after 90 min exposure to human serum and 30 min rinse in PBS (black) or 120 min exposure to only PBS (blue stripes).

## Conclusions

This study demonstrates a simple two-step approach to address the critical issues of hydrolysis and fouling in PSi-based biosensors, which are key challenges for improving their reliability and sensitivity toward measuring analytes in biological media. By thermal hydrosilylation of PSi with VBC and subsequently grafting zwitterionic polySBMA via ARGET-ATRP to its surface, we developed an antifouling, hydrolytically stable coating for PSi sensors. This surface modification achieved significant reduction in nonspecific adsorption while providing protection against hydrolysis, as evidenced by optical reflectance measurements and ATR-FTIR spectroscopy. The versatility of the hydrosilylation-ATRP approach on PSi provides a customizable platform for introduction of various capture agents for biosensing, offering promise for sensitive and selective sensing in complex biological media.

## Supplementary Material



## Notes

ATR-FTIR peak integration; ATR-FTIR of PSi hydrosilated at 120 °C before and after attempted ARGET-ATRP; reflectance spectra and images of PSi hydrosilated at 120 °C for 2 and 16 h; ATR-FTIR spectra of PSi-VBC and PSi-VBC-polySBMA following serum exposure (PDF)
