# Supplementary material for: Baseline Stability of Thermally Hydrosilated Porous Silicon with Zwitterionic Antifouling Polymer Coating for Biosensing Applications
Source: ACS Omega. 2025 Jul 16;10(29):31932–9. doi: 10.1021/acsomega.5c03495 (PMC12311861; doi:10.1021/acsomega.5c03495)
Supplement: Supplementary file 1 [file ao5c03495_si_001.pdf]

Supporting Information

BASELINE STABILITY OF THERMALLY HYDROSILATED POROUS SILICON WITH ZWITTERIONIC ANTI-FOULING POLYMER COATING FOR BIOSENSING APPLICATIONS

*Soren M. Smail[1], Paul E. Laibinis[1,2ψ], Sharon M. Weiss[1,3]**

[1]Interdisciplinary Materials Science Graduate Program, Vanderbilt University, Nashville, Tennessee 37235 United States
[2]Department of Chemical & Biomolecular Engineering, Vanderbilt University, Nashville, Tennessee 37235 United States
[3]Department of Electrical and Computer Engineering, Vanderbilt University, Nashville, Tennessee 37235 United States

[ψ]Email: paul.e.laibinis@vanderbilt.edu
*Email: sharon.weiss@vanderbilt.edu

Table of Contents

1. ATR-FTIR peak integration……………………………………………………………………………………………S2
2. Reflectance spectra and images of PSi hydrosilated at 120 °C for 2 h and 16 h……………………………………S3
3. ATR-FTIR of PSi hydrosilated at 120 °C before and after attempted ARGET-ATRP……………………………S4
4. ATR-FTIR spectra of PSi-VBC and PSi-VBC-polySBMA following serum exposure……………………………S5

1. ATR-FTIR peak integration

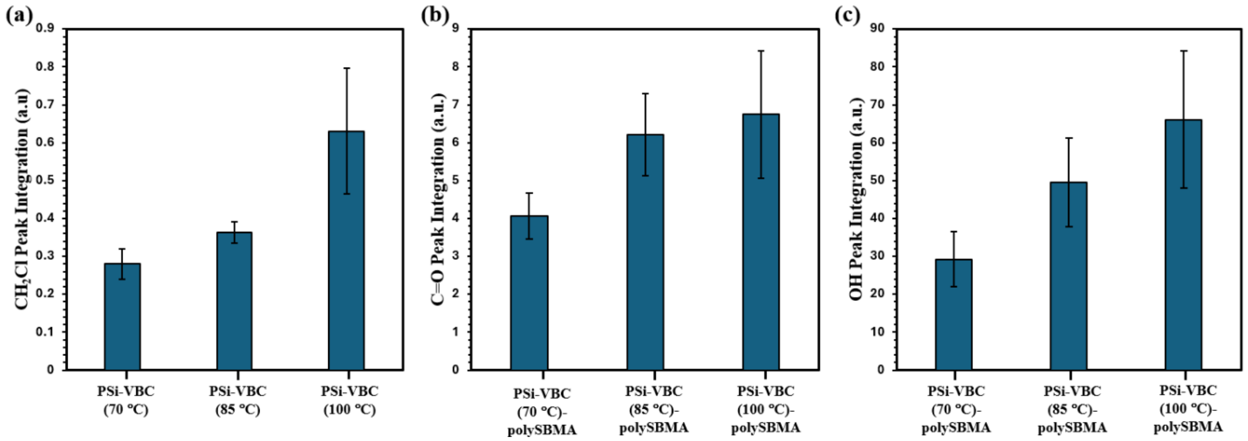

Figure S1. Average area ± one standard deviation of ATR-FTIR peaks of interest following modification of PSi surface by VBC and polySBMA. Spectra were normalized by setting their respective maximum peak intensity (near 1020 cm^{-1}) to 1. (a) For PSi hydrosilated with VBC at various temperatures, the average CH_2Cl peak area for each sample set is displayed. (b) For PSi hydrosilated with VBC at various temperatures and then further modified with polySBMA, the average C=O peak area for each sample set is displayed. (c) For PSi-VBC-polySBMA samples, the average OH peak area for each sample set is displayed.

2. Reflectance spectra and images of PSi hydrosilated at 120 °C for 2 h and 16 h

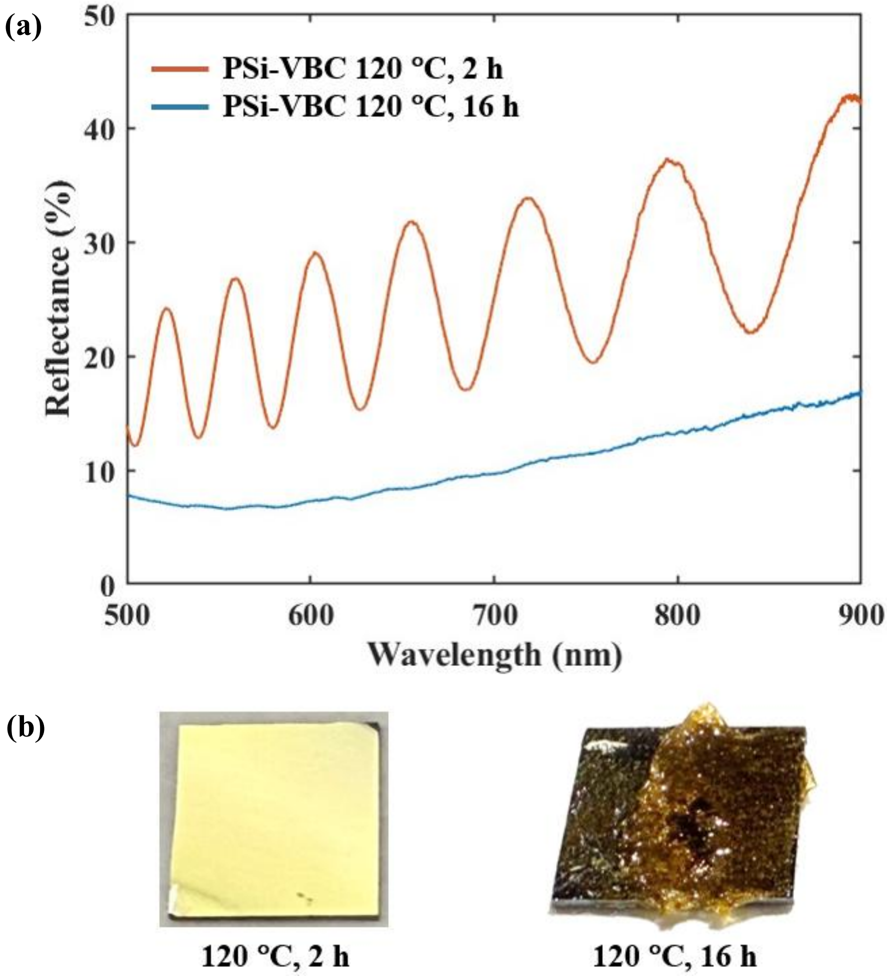

Figure S2. (a) Reflectance spectra of PSi hydrosilated with VBC at 120 °C for 2 h (orange) and 16 h (blue). (b) Pictures of the resulting functionalized PSi slides. Hydrosilylation of PSi at 120 °C in VBC for 2 h (left) retained the appearance of PSi whereas for 16 h (right) resulted in a visibly thick coating on these samples, which appeared as a gel-like substance before drying.

3. ATR-FTIR of PSi hydrosilated at 120 °C before and after attempted ARGET-ATRP

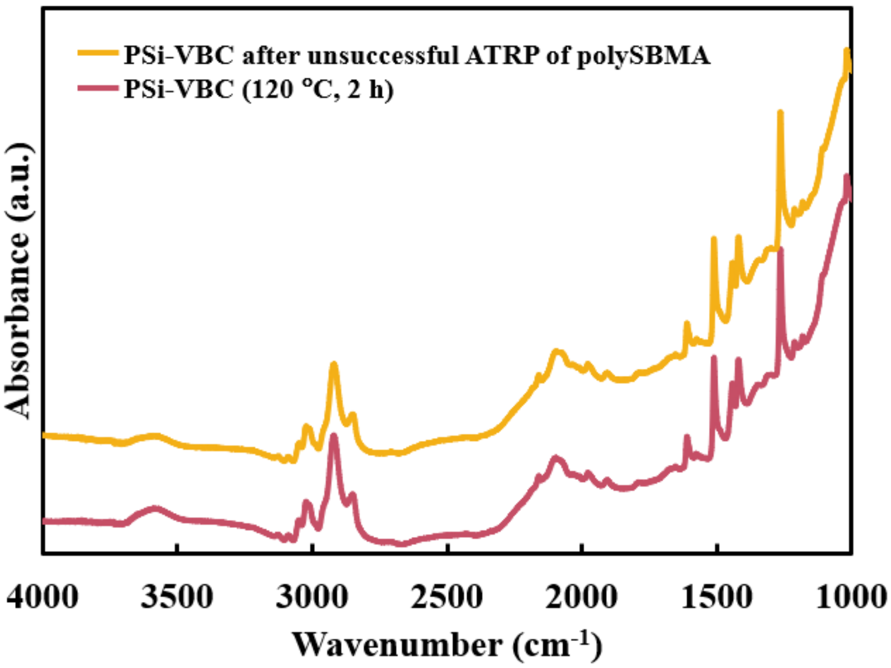

Figure S3. ATR-FTIR spectra of PSi-VBC (120 °C, 2 h) before (red) and after (orange) attempted grafting of polySBMA. Spectra have been offset vertically for clarity. Despite the observation of a peak due to the benzyl chloride moiety for PSi hydrosilated with VBC at 120 °C, the orange spectrum shows a lack of peaks for poly(SBMA) present in Figure 2b for PSi hydrosilated with VBC at lower temperatures that underwent the same exposure to ATRP conditions with SBMA.

4. ATR-FTIR spectra of PSi-VBC and PSi-VBC-polySBMA following serum exposure

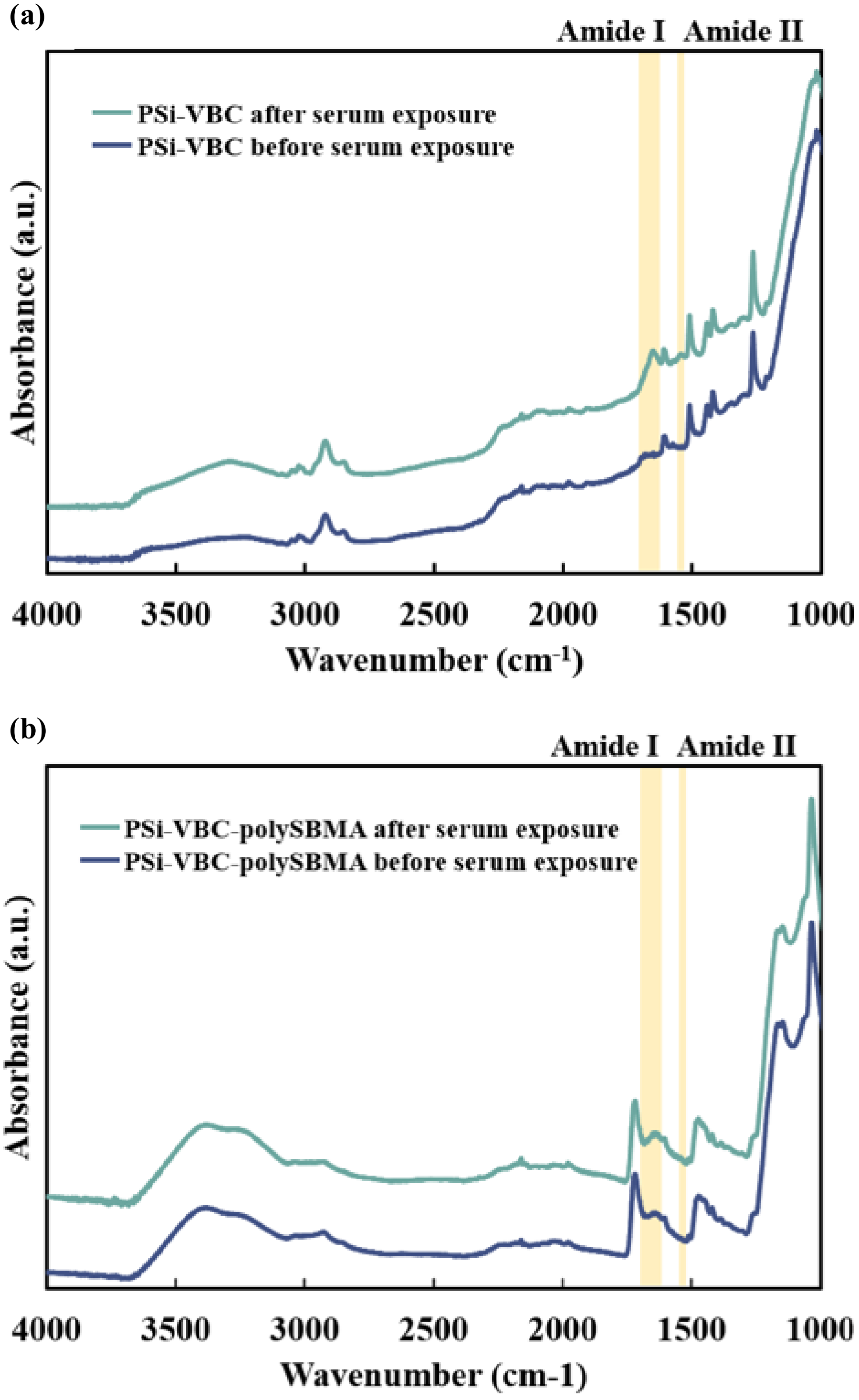

Figure S4 ATR-FTIR spectra of PSi (a) hydrosilated with VBC and (b) hydrosilated with VBC and coated with polySBMA, both before and after serum exposure. Spectra are offset vertically for clarity.
